# Novel Presentation of Terminal Ileitis Associated with Secukinumab Therapy

**DOI:** 10.1155/2021/5213876

**Published:** 2021-09-28

**Authors:** Aciel Ahmed Shaheen, Ismail Hader, Zakaria Aqel

**Affiliations:** ^1^Department of Internal Medicine, William Beaumont Hospital at Royal Oak, 3535 W. Thirteen Mile Rd, Royal Oak, MI 48073, USA; ^2^Department of Diagnostic Radiology, William Beaumont Hospital at Royal Oak, 3535 W. Thirteen Mile Rd, Royal Oak, MI 48073, USA

## Abstract

Inflammatory bowel disease (IBD) and psoriasis are chronic inflammatory immune-mediated diseases. The interleukin-23- (IL23-) T helper (Th)17 pathway has been implicated in their pathogenesis, with multiple biologic therapies targeting this pathway. IL-17, the main proinflammatory cytokine produced by (TH)17, has been targeted by antibodies and IL-17 receptor blockers with favorable outcomes in treating psoriasis and psoriatic arthritis. However, their role in IBD is unpredictable as studies reported worsening of IBD with agents targeting IL-17 and rare case reports with new-onset IBD. We present a case of Crohn's-like severe terminal ileitis and worsening diverticulitis complicated by intestinal perforation requiring total parenteral nutrition shortly after being started on secukinumab.

## 1. Introduction

Secukinumab, a fully human monoclonal antibody that selectively neutralizes IL‐17A, has been shown to have significant efficacy in the treatment of moderate‐to‐severe psoriasis and psoriatic arthritis [1]. IBD and psoriasis are both chronic immune-mediated diseases that share overlapping genetic profiles [2]. Prior studies show benefits and increased rate of adverse events in IL-17A inhibition in Crohn's disease [3]. Exacerbation of IBD or new-onset IBD has been reported in rare case reports. However, causal relationships have not been established [2]. Psoriasis with concomitant psoriatic arthritis is associated with an increased risk of Crohn's disease [4], which poses the question of whether the emergence of IBD in psoriasis patients treated with IL-17A antibodies is related to IL-17A inhibition or merely their increased risk of developing IBD.

## 2. Case Report

A 39-year-old male with a past medical history of psoriatic arthritis on secukinumab for three months PTA, previously on adalimumab, and diverticulitis presented with diffuse abdominal pain, nausea, constipation, and vomiting for three days. He had periodic bloody bowel movements 2 months following SEC induction but denied current blood or mucus in stool on admission; he reported similar abdominal pain during the loading dose of secukinumab. He denied any family history of inflammatory bowel disease.

On admission, vital signs were stable. Physical examination revealed lower abdominal tenderness with voluntary guarding, moderately distended abdomen, and normal bowel sounds. Routine laboratory tests on admission revealed a hemoglobin of 16.4 g/dL, white blood count 15.5 bil/L, platelets 401 bil/L, CRP 185 mg/dL, and ESR 50 mm/hr. Alkaline phosphatase, aspartate aminotransferase, alanine aminotransferase, lactic acid, and lipase were unremarkable. *Clostridium difficile* and stool cultures were negative.

**Figure 1:**
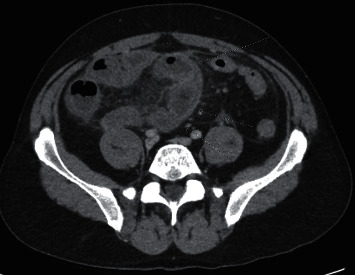
Axial CT image with IV contrast demonstrating inflammatory changes of the distal ileum. The bowel wall is thickened and edematous with surrounding fat stranding.

**Figure 2:**
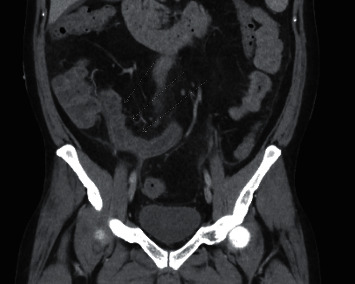
Coronal CT image with IV contrast demonstrating inflammatory changes of the terminal ileum which has a thickened wall.

**Figure 3:**
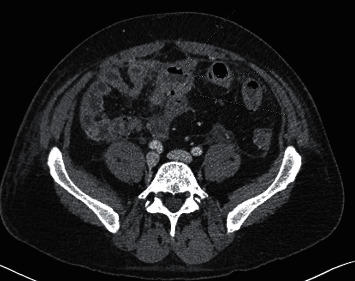
Axial CT enterography image of the lower abdomen shows persistent inflammation of a short segment of the distal ileum. This segment of the ileal wall is thickened and edematous with minimal surrounding edema.

**Figure 4:**
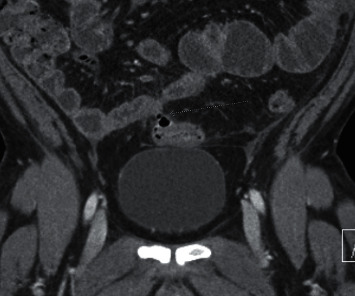
Coronal CT enterography image of the lower abdomen demonstrates a sinus tract adjacent to the extraluminal collection which bridges an air-filled sigmoid diverticulum and the adjacent distal ileal loop.

Computerized tomography of the abdomen and pelvis with IV and oral contrast demonstrated distal small bowel with severe wall edema, hyperemia, and thickening with marked surrounding inflammation, causing more proximal small bowel dilation and loops of sigmoid closely approximated with distal ileal loops setting the stage for fistulous formation and noninflamed diverticulosis in the distal colon (Figures 1 and 2). Computerized tomography enterography performed shortly after admission revealed sigmoid colon diverticulitis with a contained serpiginous air and fluid collection measuring 2.9 × 0.7 cm, which extends from the sigmoid colon's surface courses along the distal ileum, with improved distal ileal inflammation (Figures 3 and 4). A subsequent (CT) showed interval improvement in sigmoid diverticulitis and distal ileitis, with a single pocket of extraluminal air remaining and resolution of previously seen fluid collection.

**Figure 5:**
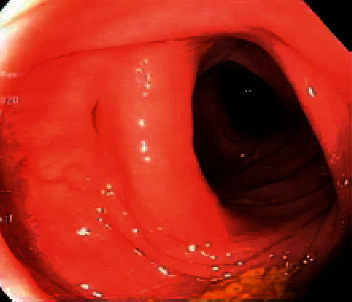
Sigmoid colon showing mild diverticulosis.

The patient was started on ciprofloxacin and metronidazole, later switched to piperacillin-tazobactam, and received total parenteral nutrition due to inability to tolerate a clear liquid diet. The patient's abdominal pain improved, and he was able to tolerate a soft diet. He was discharged on ampicillin/sulbactam to complete a 14-day course of antibiotics, to stop secukinumab, and to follow-up after four weeks for a colonoscopy which showed diverticulosis of the sigmoid colon (Figure 5) and no evidence of Crohn's disease.

## 3. Discussion

IBD, including Crohn's disease and ulcerative colitis, is characterized by chronic relapsing intestinal inflammation. Both environmental and genetic factors play a role in the pathogenesis of IBD. The interleukin-23- (IL23-) T helper (Th)17 pathway regulated by multiple genes, including IL12B and IL23R, has been associated with both IBD, psoriasis, and psoriatic arthritis [5–8]. IL23 promotes the differentiation and expansion of the effector Th17, which subsequently secretes IL-17, a proinflammatory cytokine that has been implicated in multiple autoimmune diseases [9, 10]. IL-17 expression in the intestinal mucosa of IBD patients compared to its absence in normal intestinal mucosa and the associated increase in serum has been reported in several studies, concluding that IL-17 plays a proinflammatory role in the pathogenesis of IBD [11]. In the skin, Th-17 cells mediate acanthosis, hyperkeratosis, and parakeratosis, in addition to the synthesis of inflammatory molecules within the dermis and epidermis [12].

The prevalence of IBD in psoriasis patients is 1-2% compared to 0.4% in the population [13]. Several agents, including adalimumab and infliximab, are used to treat both psoriasis and IBD. Secukinumab has been used to treat moderate to severe psoriasis. However, a study conducted to evaluate the efficacy of secukinumab in treating Crohn's disease concluded that it was ineffective and was associated with an increased rate of adverse events [3]. Brodalumab, an IL-17 receptor antagonist, was associated with worsening Crohn's disease and no meaningful efficacy [14]. A long-term safety analysis from pooled clinical trials and postmarketing surveillance of secukinumab for up to 5 years in the treatment of psoriasis and psoriatic arthritis reported the incidence of IBD was 0.01 and 0.05, respectively [15].

The role of IL-17 in the pathogenesis of dextran sodium sulfate colitis has been challenged in animal studies. Neutralization of IL-23 was associated with decreased colitis in animal studies which is clinically applicable using ustekinumab to treat TNF-resistant Crohn's disease. On the contrary, the neutralization of IL-17 was associated with the exacerbation of colitis. It was reported that IL-17 regulates occludin, the tight junction protein that limits permeability and maintains barrier integrity [16], suggesting both a proinflammatory and protective role in the gut mucosa.

To our knowledge, this is the first case of Crohn's-like severe terminal ileitis causing partial intestinal obstruction and diverticulitis complicated by intestinal perforation requiring total parenteral nutrition and antibiotics, three months after starting secukinumab. It remains unclear whether secukinumab induced Crohn's-like illness or if subclinical IBD was uncovered by stopping adalimumab or the worsening of diverticulitis caused by the inhibition of IL-17A may have a role in protecting the integrity of the gut mucosal barrier. Although casualty has not been established yet, physicians must be aware of the possible new-onset IBD following IL-17 inhibitor therapy initiation, and a careful history of gastrointestinal symptoms and family history of IBD should be obtained for monitoring these patients for the development of gastrointestinal symptoms.

## References

[1] Bissonnette R., Luger T., Thaçi D. (2018). Secukinumab demonstrates high sustained efficacy and a favourable safety profile in patients with moderate-to-severe psoriasis through 5 years of treatment (SCULPTURE Extension Study). *Journal of the European Academy of Dermatology and Venereology*.

[2] Fieldhouse K. A., Ukaibe S., Crowley E. L., Khanna R., O’Toole A., Gooderham M. J. (2020). Inflammatory bowel disease in patients with psoriasis treated with interleukin-17 inhibitors. *Drugs in Context*.

[3] Hueber W., Sands B. E., Lewitzky S. (2012). Secukinumab, a human anti-IL-17A monoclonal antibody, for moderate to severe Crohn’s disease: unexpected results of a randomised, double-blind placebo-controlled trial. *Gut*.

[4] Li W.-Q., Han J.-L., Chan A. T., Qureshi A. A. (2013). Psoriasis, psoriatic arthritis and increased risk of incident Crohn’s disease in US women. *Annals of the Rheumatic Diseases*.

[5] Zhang X.-J., Huang W., Yang S. (2009). Psoriasis genome-wide association study identifies susceptibility variants within LCE gene cluster at 1q21. *Nature Genetics*.

[6] Duerr R. H., Taylor K. D., Brant S. R. (2006). A genome-wide association study identifies IL23R as an inflammatory bowel disease gene. *Science*.

[7] Kobayashi T., Okamoto S., Hisamatsu T. (2008). IL23 differentially regulates the Th1/Th17 balance in ulcerative colitis and Crohn’s disease. *Gut*.

[8] Cho J. H. (2008). The genetics and immunopathogenesis of inflammatory bowel disease. *Nature Reviews Immunology*.

[9] McGeachy M. J., Chen Y., Tato C. M. (2009). The interleukin 23 receptor is essential for the terminal differentiation of interleukin 17-producing effector T helper cells in vivo. *Nature Immunology*.

[10] Langrish C. L., Chen Y., Blumenschein W. M. (2005). IL-23 drives a pathogenic T cell population that induces autoimmune inflammation. *Journal of Experimental Medicine*.

[11] Fujino S., Andoh A., Bamba S. (2003). Increased expression of interleukin 17 in inflammatory bowel disease. *Gut*.

[12] Skroza N., Proietti I., Pampena R. (2013). Correlations between psoriasis and inflammatory bowel diseases. *BioMed Research International*.

[13] Eppinga H., Poortinga S., Thio H. B. (2017). Prevalence and phenotype of concurrent psoriasis and inflammatory bowel disease. *Inflammatory Bowel Diseases*.

[14] Targan S. R., Feagan B., Vermeire S. (2016). A randomized, double-blind, placebo-controlled phase 2 study of brodalumab in patients with moderate-to-severe Crohn’s disease. *American Journal of Gastroenterology*.

[15] Deodhar A., Mease P. J., McInnes I. B. (2019). Long-term safety of secukinumab in patients with moderate-to-severe plaque psoriasis, psoriatic arthritis, and ankylosing spondylitis: integrated pooled clinical trial and post-marketing surveillance data. *Arthritis Research and Therapy*.

[16] Lee J. S., Tato C. M., Joyce-Shaikh B. (2015). Interleukin-23-independent IL-17 production regulates intestinal epithelial permeability. *Immunity*.

